# Extreme Energy Dissipation via Material Evolution in Carbon Nanotube Mats

**DOI:** 10.1002/advs.202003142

**Published:** 2021-01-29

**Authors:** Jinho Hyon, Olawale Lawal, Ramathasan Thevamaran, Ye Eun Song, Edwin L. Thomas

**Affiliations:** ^1^ Department of Materials Science and NanoEngineering Rice University Houston TX 77005 USA; ^2^ Department of Materials Science and Engineering Texas A&M University College Station TX 77843 USA; ^3^ Department of Chemistry United States Air Force Academy El Paso CO 80840‐5002 USA; ^4^ Department of Engineering Physics University of Wisconsin‐Madison Madison WI 53706 USA

**Keywords:** carbon nanotube mats, extreme energy absorption, high strain rate deformation, laser induced projectile impact test

## Abstract

Thin layered mats comprised of an interconnected meandering network of multiwall carbon nanotubes (MWCNT) are subjected to a hypersonic micro‐projectile impact test. The mat morphology is highly compliant and while this leads to rather modest quasi‐static mechanical properties, at the extreme strain rates and large strains resulting from ballistic impact, the MWCNT structure has the ability to reconfigure resulting in extraordinary kinetic energy (KE) absorption. The KE of the projectile is dissipated via frictional interactions, adiabatic heating, tube stretching, and ultimately fracture of taut tubes and the newly formed fibrils. The energy absorbed per unit mass of the film can range from 7–12 MJ kg^−1^, much greater than any other material.

## Graphical abstract

Porous, unoriented multiwall carbon nanotube mats dissipate extraordinary amounts of kinetic energy by a variety of processes via their ability to dramatically reconfigure, align, and strengthen during shock impact to exhibit the highest specific energy absorption (≈12 MJ kg^−1^) for projectile perforation of any known material.

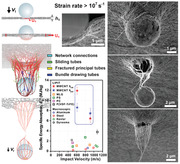

## 1 Introduction

Lightweight, flexible materials for military personnel and for aerospace structural protection are often made from high modulus, high strength polymer fibers.^[^ ^1^ ^]^ Their mechanical behavior depends on the intrinsic polymer fiber properties as well as the interactions between fibers.^[^ ^2^ ^]^ Due to their inherent low densities and outstanding mechanical properties, carbon nanotube (CNT) materials are very attractive for applications where performance per unit weight is critical.^[^ ^3^ ^]^ Previous experiments and theoretical predictions^[^ ^4^, ^5^, ^6^ ^]^ indicate that individual, perfectly aligned, defect‐free, single wall (SW) and multiwall (MW) CNTs could exhibit tensile modulus of 1 TPa, strengths > 100 GPa, with failure strains of 5–15%. Here we show that, contrary to expectations, initially low modulus, low strength high porosity MWCNT mats comprised of initially unaligned, individual tubes and tube bundles bonded together into networks by both covalent and secondary bonds can dissipate unprecedented amounts of kinetic energy (KE) per unit mass by dramatic changes in both the mat morphology and in the CNT–CNT interactions occurring during the impact‐perforation event. These include mat compaction, tube collapse, alignment, fibril formation, and ultimately tensile fracture of taut bundles and newly formed fibrils.

Experimental values of modulus, fracture strength, and failure strain of CNTs cover a wide range depending whether the test is on a SW or MW individual tube, tube bundle or fiber, the length of the specimen tested as well as the type of processing used.^[^ ^4^, ^7^, ^8^, ^9^, ^10^, ^11^ ^]^ Collapsed MWCNTs exhibit increased strength due to increased contact areas^[^ ^7^, ^12^ ^]^ and MWCNT fibers have been produced having quasi‐static mechanical properties with a toughness (energy required for failure) that can be superior to commercial high performance fibers (Table S1, Supporting Information).^[^ ^9^ ^]^ However, production of SWCNT fibers is limited to lab scale spinning and both SW and MW fibers fall well short of their predicted strengths because of relatively short tube lengths and the low interfacial interactions with failure occurring due to tube pull out.^[^ ^6^ ^]^ Interestingly, while a single tube or aligned bundle exhibits linear elastic behavior, yarns of MWCNTs are highly hysteretic for loading/unloading,^[^ ^13^ ^]^ due to stress relaxation from sliding between tubes. Significantly enhanced fracture toughness was noted for aligned MWCNT mats made from vertically grown CNT forest precursors for tensile loading parallel to the alignment direction (crack propagation perpendicular to the alignment direction).^[^ ^14^ ^]^ The large fracture toughness is attributed to the inherent high strength of the CNTs, and the strong tube–tube interactions due to the extensive van der Waals forces acting between highly aligned and axially overlapped tubes resulting in a tortuous crack path due to crack bifurcations along with frictional hysteresis from tube sliding in a dissipative zone ahead of the crack tip.

Recently high velocity lateral impact tests on a series of high‐performance fibers including solution spun SWCNT fibers showed that SWCNT fiber absorbed more impact energy than the other fibers, attributed to strain rate amplified friction in the SWCNT material.^[^ ^15^ ^]^ These characteristics of CNT fibers raise the notion that a highly dissipative, yet ultimately stiff and strong material could prove useful as a protection material.

We investigate planar isotropic MWCNT porous mats made by Tortech Nano Fibers using a gas phase catalytic reaction that employs a floating metal catalyst to nucleate and grow extremely long tubes (millimeters).^[^ ^16^ ^]^ The process creates an aerogel‐like network of tubes and tube bundles (also known as “sock”) that is continuously removed from the reaction zone. The aerogel sock is collapsed onto a rotating drum to build up a mat consisting of many thin layers of tubes having tortuous contours but relatively few 3D entanglements. For a low take up speed, the tubes can be nearly unoriented. The quasi‐static uniaxial tensile properties of a similar unoriented Tortech mat material revealed very modest properties compared with those from individual tubes with in plane tensile modulus of only ≈3 GPa, tensile strength of 30–40 MPa with the strain to failure around 20–30%.^[^ ^17^ ^]^

## 2 Results and Discussion

**Figure 1:**
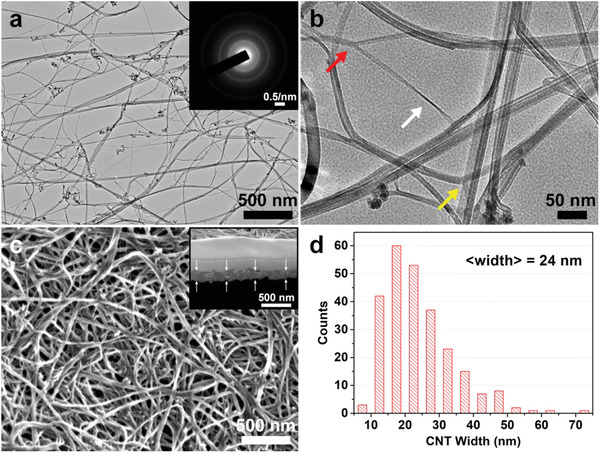
MWCNT mat morphology a) BFTEM image of the single sock aerogel showing tubes, bundles, and tube–tube junctions. Inset: electron diffraction pattern. b) Higher magnification BFTEM image of Y (red arrow) and T (yellow arrow) type tube–tube network junctions. Most of the MWCNTs are collapsed (e.g., white arrow: bundle width changes due to twisting of the collapsed tubes). c) SEM image of the MWCNT mat. Inset: FIB cross sectional 52° tilted SEM view of an MWCNT film with a Pt–C protective top layer with an average film thickness *h*_205_ ≈ 205 ± 12 nm. d) The TEM width distribution of MWCNT tubes and tube bundles with number average of 24 ± 10 nm.

Bright field transmission electron microscope (BFTEM) images of a single layer of the aerogel sock show a highly porous interconnected bundle network (**Figure** **1**a–c). Following along the contour of a given tube or tube bundle shows shape transitions from wide to narrow to wide etc., indicative of twisted, collapsed‐flattened multiwall tubes. Collapsed MWCNTs are observed to bond along their axial direction to form larger bundles and these bundles join, branch apart (Y junctions), re‐join, and fuse to other tubes/bundles with load transfer by both covalent bonds^[^ ^11^ ^]^ (T junctions) and van der Waals interactions (Figure 1b; Figure S1, Supporting Information). SEM images (Figure 1c) show long, meandering, variable width tubes, and tube bundles (Figure 1d). Void regions between tube bundles are also evident in cross sectional scanning electron microscope (SEM) images made by focused ion beam (FIB) milling (Figure S2a, Supporting Information). The small angle and wide angle X‐ray scattering patterns (SAXS, WAXS) for the beam incident normal to two pieces of the 42 micron thick mat (Figure S2c,d, Supporting Information) as well as for the electron diffraction (ED) pattern of an ultrathin peeled layer (≈100 nm) (Figure 1a inset) exhibit isotropic 2D low angle scatter (SAXS) and a near‐constant azimuthal intensity of the (002) Bragg peak (ED, WAXS), consistent with an approximate 2D isotropic distribution of the tube axes in the plane of the film.

The laser induced projectile impact test (LIPIT) has been used to investigate ballistic energy absorption for thin film materials, including multilayer graphene,^[^ ^18^ ^]^ and glassy polymers.^[^ ^19^ ^]^ In LIPIT (Figure S3, Supporting Information), a laser pulse is used to launch a small projectile (e.g., 3.7 µm diameter silica sphere) to impact a target specimen resulting in initial shock compression, followed by axisymmetric tensioning, shear localization, and ultimately perforation. The projectile velocity is measured before impacting and just after perforating the target to determine the loss in translational kinetic energy (ΔKE). To understand how thin portions of the MWCNT mats deform, we explored a wide range of mat thicknesses and projectile velocities. We elaborate our analysis on the *h*_205_ = 205 ± 12 nm samples since at this mat thickness for our chosen set of impact velocities of *v*_305_ = 305 ± 4, *v*_611_ = 611 ± 7, and *v*_916_ = 916 ± 10 m s^−1^, projectiles are arrested at the lowest velocity and all projectiles perforate at the highest velocity. Impacting at velocities below, near, and above the perforation threshold allows the observation of a range of deformation modes as well as estimation of the V50 ballistic limit for the target material.

**Figure 2:**
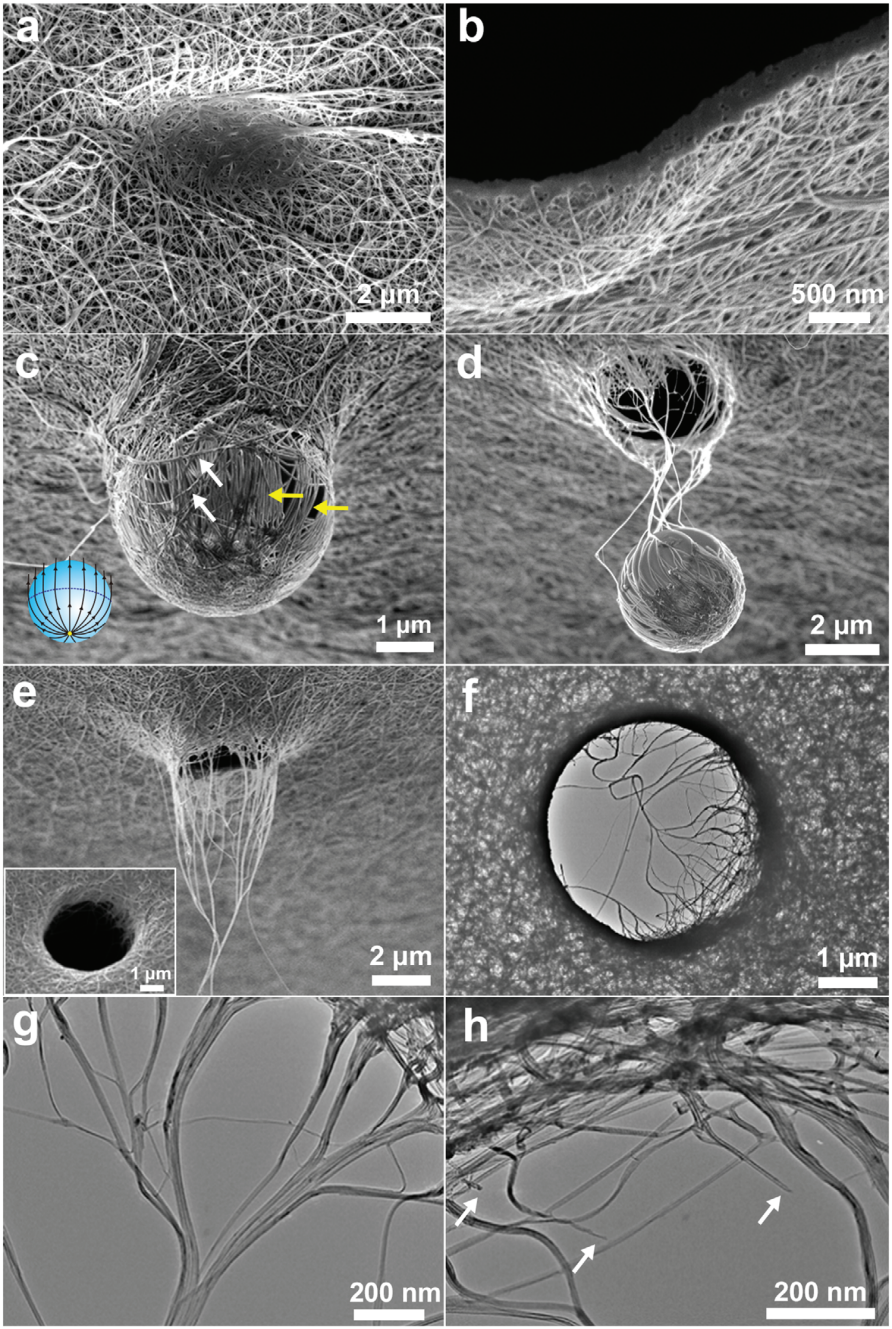
Morphological and material evolution during projectile shock compression, penetration, and perforation a–e) SEM images (tilted 52°) of backside of film (except (a)) and f–h) TEM images. a) Indentation region of front surface showing compaction/fusion of mat in center. b) FIB cross section showing compaction/densification of the mat under the projectile. c) SiO_2_ projectile arrested at *v*_611_ showing *h*_205_ mat deformation with alignment of principal tubes (yellow arrows). Non‐principal tubes are observed to slide to the side of the projectile (white arrows). Inset: Schematic of longitudinal principal (load carrying) tubes. d) Arrested *v*_611_ sphere still connected to the *h*_205_ mat with compacted film in the impact region surrounded by highly drawn, extended tubes/fibrils. e) Perforated *h*_205_ mat impacted at *v*_611_. Inset: View of perforation from impact side. f–h) BFTEM images (normal views) for *v*_611_ and *h*_205_ mat. g,h) Higher magnification images illustrating the drawing of tubes and bundles into fibrils from the peripheral region. White arrows in (h) indicate fractured tube ends.

At *v*_305_, the mat stops all the projectiles. Upon impact, a shock wave propagates through the mat, compacting the mat and adiabatically heating the tubes. Estimated strain rates are from 10^7^–10^10^ s^−1^ with near instantaneous increases in temperature and pressure.^[^ ^20^ ^]^ SEM of an impact region on the mat front side shows smooth, collapsed, and fused regions at the center (**Figure** **2**a). A FIB cross section through an arrested sphere shows the film thickness has decreased by about a factor of 2 (Figure 2b). As the sphere penetrates, certain tubes in the central impact region, termed principal tubes (yellow arrows in Figure 2c) begin carrying load and straighten by slipping across the projectile surface and past their partially loaded neighbors. Tubes further from the impact center slide to the sides of the sphere (white arrows). The principal tubes become extended along longitudinal lines on the sphere surface (Figure 2c inset scheme), emanating outward from the top of the sphere and the deformation texture appears approximately axisymmetric. Occasionally (≈25%), we observe the appearance of latitudinal wrapped tubes (oriented orthogonal to the longitudinal tube directions), suggesting that the particular impacting sphere was rotating at a high angular velocity and carrying additional rotational KE (Figure S4, Supporting Information).

For the mid‐range velocity, *v*_611_, about 70% of the projectiles perforate the film, (see Figure 2c–e; Figure S4e, Supporting Information). TEM images of perforations (Figure 2f–h) allow insight on how forces transmitted from the principal tubes moving with the projectile serve to translate, bring together, and align tubes via a type of local “fiber spinning” process^[^ ^13^ ^]^ resulting in the formation of larger diameter, stronger, load bearing fibril elements. Due to the extensive branching of the tube network, the load is distributed radially outward. SEM images show straightened and fractured tubes/fibrils extending tens of microns from the exit surface. Captured SiO_2_ projectiles show fractured tubes wrapped over the surface but without any apparent cracking or shape deformation of the projectile (Figures S4d and S5, Supporting Information). The projectile undergoes shock loading and adiabatic heating but we neglect this contribution to energy dissipation in the projectile during impact. At *v*_916_, there is complete perforation of the film for every shot (Figure S6, Supporting Information) and the KE absorbed is lower than for *v*_611_.

**Figure 3:**
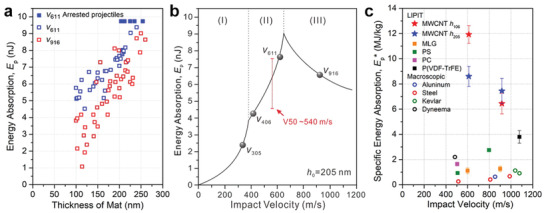
Energy absorption versus mat thickness and incident velocity. a) Kinetic energy (KE) absorption versus mat thickness for two incident velocities. Solid blue squares denote arrested projectiles at *v*_611_. b) Trends of energy absorption as a function of impact velocity for a specimen of a given thickness indicating how our measurements fit within the 3 regimes (I, II, and III) of energy absorption as a function of projectile velocity: I) below the ballistic limit, II) above the ballistic limit, and III) high‐velocity regime.^[^ ^21^ ^]^ The V50 value (Figure S8, Supporting Information) occurs in regime (II). c) Specific energy absorption (*E*_p_^*^) values (in MJ kg^−1^) obtained from (a) along with prior best *E*_p_^*^ data from LIPIT and literature data from macroscopic ballistic tests of various materials with comparable ratios of the projectile diameter to membrane thickness. *E*_p_^*^ values of MWCNT mats show *h*_205_ at *v*_611_ = 8.59 ± 0.30 MJ kg^−1^ (*n* = 15 shots), *h*_205_ at *v*_916_ = 7.44 ± 1.03 MJ kg^−1^ (*n* = 13), *h*_106_ at *v*_611_ = 11.92 ± 0.74 MJ kg^−1^ (*n* = 13), and *h*_106_ at *v*_916_ = 6.44 ± 0.82 MJ kg^−1^ (*n* = 12). Other details of specimens and testing conditions are available in Table S2, Supporting Information.

The change in the translational KE required to perforate the MWCNT mats, *E*_p_, can be directly calculated by measuring the incident and residual projectile velocities while accounting for the KE lost from air drag and film motion (see Supporting Information). Rotational KE can contribute small additional energy dissipation, but we base our determination of the absorbed KE only on translational velocities (see Supporting Information). Thus, *E*_p_ = 1 / 2(*m*_p_ *v*_i_^2^ – (*m*_p_ + *m*_plug_) *v*_r_^2^) + *E*_drag_ where *E*_drag_ is the energy loss due to drag, *m*_p_ is the projectile mass (kg), and *m*_plug_ is the mass of the target adhering to the projectile after penetration, *v*_i_ is the impact velocity (m s^−1^), and *v*_r_ is the residual velocity. Use of a capture plate is important to determine the amount (if any) of target material adhering to the projectile after perforating. In the case of MWCNT mats, the mass of the plug is only about 1% to that of the sphere, so this correction is small. **Figure** **3**a shows the corrected KE loss as a function of film thickness for both *v*_611_ and *v*_916_ with the values decreasing for *v*_916_.

For lightweight applications, a key figure of merit is the specific energy absorption, $E_{p}^{*}$. This addresses the ability of the material to absorb energy relative to the mass of the material in the minimum target plug region and is defined as $E_{p}^{*}=E_{p}\;/\;m_{\mathrm{plug}}\;$, where *m*_plug_ is the mass of the target plug, given by the product of the projectile strike face area and original film thickness and density (see Supporting Information). $E_{p}^{*}$ for the MWCNT mats using data from Figure 3a is plotted in Figure 3b,c. We also include the values of $E_{p}^{*}$ from the literature for macroscopic spherical projectiles perforating thin membranes (with *D*/*h*_0_ > 4) and the highest reported values from prior LIPIT research on ultrathin films (Figure 3c; Table S2, Supporting Information). At *v*_611_, the 106 nm thick MWCNT mats exhibit an $E_{p}^{*}$ value of ≈12 MJ kg^−1^, larger than any LIPIT measurements and 5 to 14 times that of Dyneema/Kevlar (Table S2, Supporting Information) impacted with macroscopic projectiles. In Figure 3b, the drop off of the absorbed energy at the highest velocity occurs due to the shorter time scale of the perforation process at the highest velocity and reflects the onset of the inability of the tubes in the peripheral region to reconfigure and strengthen the mat by pull‐in and alignment during the shorter impact event.

## 3 Conclusion

**Figure 4:**
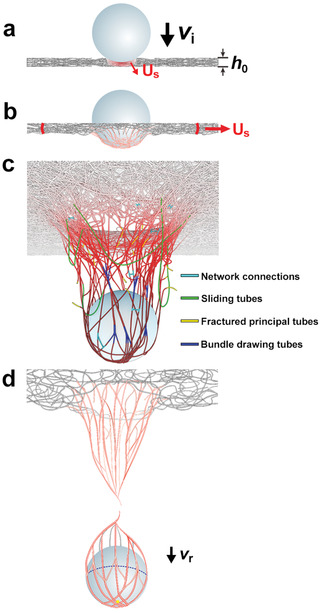
Morphological and material evolution during perforation. a) Sphere with incident velocity *v*_i_ impacts porous mat with initial thickness *h*_0_. The compressive shock wave with velocity (*U*_s_) causes mat densification and adiabatic heating. b) Principal tubes (red color) translate, straighten, and align along longitudinal directions, distributing the load radially outward. c) Tubes elongate and draw together to form strong fibrils. d) The speed of the sphere has decreased to a residual velocity *v*_r_ due to the retarding forces which eventually reach the tensile fracture stress of principal tubes/bundles/fibrils.

The remarkably high values of $E_{p}^{*}$ from a material with low quasi‐static tensile properties ^[^ ^17^ ^]^ are unexpected. While individual carbon nanotubes and tube bundles can exhibit very high intrinsic mechanical properties if efficiently packed and well aligned,^[^ ^4^, ^8^ ^]^ due the high porosity and 2D meandering nature of the tubes and bundles in these mats, these mechanical attributes of stiffness and strength are not in play at the early stages of loading but because of the strong morphological evolution, become highly influential just prior to projectile arrest/perforation. This suggests that the extraordinary $E_{p}^{*}$ values of the MWCNT mats originate from a distinctive set of morphology‐material dependent deformation‐toughening and strengthening mechanisms. **Figure** **4**a–d schematically illustrates the sequence of key transformations envisioned during impact and mat perforation. Initially, the shock impact compresses, densifies, and adiabatically heats the film, however, due to the temperature insensitivity of strength and modulus, the material does not thermally soften, rather as the frictional forces develop between the surface of the moving sphere and the tubes, the resultant stresses serve to translate, straighten, and align the principal tubes and as the sphere continues forward, the elongational deformation induces lateral coalescence of tubes and bundles at the sides and behind the sphere creating larger diameter, well aligned, strong, stiff fibrils that either slide off the projectile or undergo tensile fracture. Of interest are questions concerning which of the many energy dissipation mechanisms are dominant at the various stages of the impact, how to optimize the tradeoff of tube orientation, packing, and porosity on initial properties versus ability of the mat to transform its structure at extreme rates and high temperatures, and how the particular energy absorbing mechanisms of the material scale with size and mass of the projectile and with tube length, mat thickness, and degree of orientation.

## 4 Experimental Section

##### Preparation of Thin Mats

In order to create mats that could be perforated by the silica microprojectiles, a derivative of the Lee et al. method^[^ ^18^ ^]^ was used for making multi‐layer graphene thin films from millimeter thick sections of highly ordered pyrolytic graphite (Figure S7, Supporting Information). The as received Tortech Nano Fibers MWCNT 42 micron thick mat was placed on the adhesive surface of a water‐soluble tape having a water‐soluble adhesive. Subsequently a portion of the mat was peeled off to delaminate a thin layer. Mechanical exfoliation of the MWCNT films could produce 100–300 nm thick MWCNT specimens as measured from SEM imaging of focused ion beam cross sections. Next, an organic based liquid adhesive (Scotch Super^TM^ 77, 3M) was diluted in toluene (≈10 wt%) and applied to a nickel TEM grid to coat the grid frame with a non‐water soluble adhesive. Before drying, air was blown through the grid holes to eliminate any adhesive spanning across grid holes, so as to only coat the TEM grid frame. The MWCNT/tape laminate was then mounted onto the TEM grid and placed on the surface of deionized water for 24 h. After dissolution of the water soluble adhesive/tape, the MWCNT film adhered to the TEM grid. The grid/film was then dried in vacuum at 60 °C for 24 h before LIPIT testing. Transmission optical microscopy employing a Zeiss Axioskop microscope with 20 ×, NA 0.4 was used to map out the target locations on each TEM grid before LIPIT testing to avoid shooting non‐uniform thickness film areas. Finder TEM grids were used (Ted Pella, INC. M/P#: G2761N) so the deformation morphology could be correlated with the measured KE loss for each individual impact event.

##### Mat Density

The volumetric density of the as‐received mat was determined as 402 kg m^−3^ by weighing a portion of the mat having a known area and thickness determined by calipers (confirmed by SEM cross sectional images of CO_2_ laser cut mats). The volumetric density accounts for not only the empty regions within and between bundles, but also the hollow interiors of the MWCNTs. A Micromeritics Instrument Corporation AccuPyc 1340, employing a sample chamber volume of 1 cm^3^ was used to determine the bulk density of the mat. The density is ≈1450 kg m^−3^ averaged over six independent specimens with each measurement repeated 10 times. The difference between the volumetric and bulk density gave an average mat porosity (including the hollow tube interiors) of 72%. The volumetric and bulk densities were similar to the values found by Stallard et al.^[^ ^17^ ^]^ on similar Tortech mats.

##### Projectile Capture

To capture the SiO_2_ projectiles after perforation of the target films, a PS‐*b*‐PDMS film was prepared by spin‐coating onto a silicon wafer at 1500 rpm for 1 min using 10 wt% of PS‐*b*‐PDMS (24 –21 kg mol^−1^) in toluene to produce ≈5 mm diameter, 20 µm thick capture plate. Both the projectile capture plate and the TEM target grid were mounted onto opposite sides of a microscope cover glass slide using carbon tape to create ≈500 µm distance between each component. After perforation, the capture plate and projectiles were coated with 3 nm of gold and analyzed by SEM (Figures S4d and S5a, Supporting Information).

##### Wide‐Angle and Small‐Angle X‐Ray Scattering

WAXS and SAXS measurements were performed at beamline 12‐ID‐B at the Advanced Photon Source of the Argonne National Laboratory. Samples were irradiated with a X‐ray beam with energy of 13.3 keV and size of 10 × 20 µm^2^ with 1–5 s exposure times. Scattered X‐rays were collected with Pilatus2M (SAXS) and PerkinElmer (WAXS) detectors. Standard data correction procedures were applied using software programs available at the beamline. The sample was made by razor cutting two pieces of the mat along the midline of the mat and maintaining the registration of the overall direction of each layer with respect to the drum roller take up direction.

## Conflict of Interest

The authors declare no conflict of interest.

## Supporting information

Supporting Information
