# Supplementary material for: Extreme Energy Dissipation via Material Evolution in Carbon Nanotube Mats
Source: Adv Sci (Weinh). 2021 Jan 29;8(6):2003142. doi: 10.1002/advs.202003142 (PMC7967058; doi:10.1002/advs.202003142)
Supplement: Supplementary file 1 — Supporting Information [file ADVS-8-2003142-s001.pdf]

WILEY-VCH

© 2021 Wiley-VCH GmbH

ADVANCED SCIENCE
Open Access

Supporting Information

for *Adv. Sci.*, DOI: 10.1002/advs.202003142

Extreme Energy Dissipation via Material Evolution in Carbon Nanotube Mats

*Jinho Hyon, Olawale Lawal, Ramathasan Thevamaran, Ye Eun Song and Edwin L. Thomas**

Supporting Information

Extreme Energy Dissipation via Material Evolution in Carbon Nanotube Mats

*Jinho Hyon, Olawale Lawal, Ramathasan Thevamaran, Ye Eun Song and Edwin L. Thomas**

Launch Pad Preparation

The projectile launch pad was made following the procedure of Lee et al.[1] using a 22×22 mm^2 microscope cover slip (Fisherbrand 12-541-B) with 10 nm of gold coated onto the cover glass using a sputter coater (Denton Desk V Sputter System). The PDMS pad was prepared using a PDMS kit (Sylgard 184, Dow Chemical), consisting of a DMS monomer and a hardener with a 10 to 1 ratio of DMS monomer to hardener. To make a 20 μm thick PDMS top layer, the liquid was spin coated at 3000–4000 rpm using a Laurell WS-400BZ-6NPP/LITE. Residual air pockets were removed by placing the cover slips in a vacuum (Lab-Line 3628-1 Squaroid Duo-Vac Vacuum Oven) for 30 minutes. The PDMS was further cured by post baking just under 60 °C for 24 h. Silica projectiles (microParticles GmbH, 3.72 μm diameter-silica density of 1,900 kg/m^3) were suspended and diluted in ethanol solution (0.015 wt%). A 10 μL pipet was used to place about 6 drops onto the PDMS launch pad and the silica particles were spread across the surface using a lab wipe sheet (Kimberly-Clark Kimwipe).

Electron Microscopy and Electron Diffraction

From examination of the micrographs of the tubes surrounding and adhering to the projectile, we are able to draw conclusions concerning the evolution of the shapes and positions of the tubes as influenced by the penetrating sphere. Transmission electron microscopy was performed on a FEI Titan Themis TEM operated at 80 keV. Bright field images at low beam flux and dose were taken to avoid specimen damage. Such images allow measurement of the

MWCNT tube and bundle widths and the detailed network morphology of the tubes and bundles in a single sock layer and in the impacted regions of various thickness films for various incident velocities. Electron diffraction patterns of the pristine mats were obtained using a 20 micron diameter selected area diffraction aperture. SEM imaging of the uncoated MWCNT film, cross sections, deformation and perforation features was done using the FEI Helios NanoLab 660 SEM operated at 1 keV. Front and exit surface images were taken at tilts ranging from 0˚ to 52˚.

Mat Thickness Measurements

The thicknesses of the peeled MWCNT mat specimen targets were estimated from cross-sectional SEM images. Before milling, a protective layer of ~200 nm of Pt was deposited onto the mat to minimize ion beam damage. The cross section was made with a Helios 660 instrument using the focused gallium ion beam (30 keV, 7.7 pA). Two to three thickness measurements were conducted at the sides of each perforation to obtain the average local mat thickness.

Normalization of Energy Absorption

In macroscopic ballistic testing, good ballistic materials dissipate the impact energy far beyond the region defined by the projectile strike face area. But because the actual amount of target material involved in the deformation is difficult to determine (there is often a strong radial gradient of the deformation and associated energy dissipated), it is common to use the mass of the material below the strike face area for normalization/comparison for all materials investigated (Table S2).

Fracture of MWCNTs

The published experimental quasi-static uniaxial work to fracture (toughness) of MWCNT fibers[2] (made in a process similar to Tortech) is 0.12 MJ/kg, about 100× smaller than our high rate E_p^* value. Macroscopic, well aligned MWCNT fibers load elastically until failure when shear stresses cause the tubes to slide apart due to the very low inter-graphene shear strength with little to no actual tensile fracture of tubes.[3] On the contrary, the meandering collapsed tube network in the MWCNT mats dissipates energy due to many different deformation mechanisms and because of the loading geometry, importantly the specific work to fracture includes tensile fracture of principal tubes (Figures S1 and S5).

Projectile Spin

The spherical projectiles are sometimes found after perforation with with tubes wrapped latitudinal directions (Figure S4). This could arise from either having variable local mat properties such that the sphere penetrates more readily on one side or from projectile spin. For example, in the left region of Figure S4a, the projectile has deflected part of the mat forward into a dome-like shape, but at the right side, the combination of the forward and rotational motions of the sphere have thinned the mat and extended and aligned the tubes into a parallel array with the direction of tube alignment normal to the axis of rotation. Previous LIPIT testing[1, 4-6] did not address projectile spin but here the occasional distinct latitudinal wrapping by the tubes clearly implies that friction strongly couples the tubes to the silica surface and that projectiles can sometimes carry rotational KE. We crudely estimate the (initial) surface rotational velocity and hence incident rotational kinetic energy of $1/2\, I\omega^2$, where I is moment of inertia of the projectile, by using the time it takes for the sphere to be arrested by the film (event time ~(distance traveled by arrested sphere)/(incident velocity+final velocity)/2) and assuming that the average included angle of a wrapped tube over the sphere surface is ~π/2 radians. These simple assumptions lead to a quite high angular velocity of ~1.3×10^8 rad/s, with corresponding incident rotational KE of about 0.6 nJ. This

value is small compared to the incident translational KE value of 9.7 nJ at v_{611} indicating that the dissipation of some rotational KE can be a small additional contribution to the total energy absorbed. Projectile spin influences the mat deformation characteristics and potentially could act to decrease the observed v_r. By correlating the measured translational KE loss with the appearance of each perforation, it appears that spin interactions do not much influence v_r since the symmetric and asymmetric appearing perforations have very nearly equal average translational KE loss values at both v_{611} and v_{916} (Figure S4e). By analyzing the deformation textures of perforations, we estimate about 25% of the v_{611} projectiles (~13 out of 55 events) underwent rotation.

Strain Rate

The strain rate $d\varepsilon/dt$ varies greatly due to the complex microstructure of the MWCNT mat containing meandering and branched tubes resulting in differences of the loading of tubes in the impact area as well as significant densification and inwards flow of material into the deforming region. If we only consider the film to undergo 1D elastic deformation as done in previously,[1, 5] the average $d\varepsilon/dt$ can be approximated as ~10^7 s^{-1}. Computational modeling of a shock impact into a thin film for velocities in the 300–900 m/s range shows that the local strain rates at the impact site are much larger due to the size effect and thermodynamic equilibration rate behind a shock front.

Air Drag Corrections

The influence of projectile-air drag and air resistance to the movement of the target film were considered on the translational KE loss of the projectile during target perforation as previously addressed.[5] In order to correct the initially measured velocity to the velocity just before impact and the measured residual velocity to that immediately after perforation of the film, we use our previous determination of the projectile drag coefficient C_D, for different

velocities (100–1000 m/s) measured using a multiframe video camera. The impact velocity is calculated from a double-exposure image of the projectile (with no target present) and the residual velocity is calculated in a separate measurement by a second double-exposure of the projectile after target perforation. We then recheck the v_i without a target and find consistent values (e.g. 611±6 m/s). The small drag correction is applied to incident and exit velocities to give the instantaneous speeds at the target film location. Due to the high porosity of the thin MWCNT film and the relatively steep film shape during penetration/perforation, the target-air dissipation will be small and we estimate <0.06 nJ.

Fiber Type	Strength (GPa)	Stiffness (GPa)	Toughness (MJ/kg)
CNT fiber[2]	8.8	357	0.198
Kevlar KM2[7]	3.6	80	0.048
Dyneema[8]	3.6	115	0.066
HS carbon fiber*[9]	7.1	294	0.043

*Toray T1000

Table S1. Comparative mechanical data
Mechanical data for high performance fiber systems including CNT fiber and commercially available fibers. Typical fiber diameters are 5–20 μm and gauge lengths are 1–100 mm.

Sample Code	Sample information	Projectile parameters	Impact velocity range (m/s)	E_p^* range (MJ/kg)
MLG[1]	Multilayer graphene h_0=10–100 nm	Spherical silica D=3.7×10^{-6} m m=5.20×10^{-14} kg	600–900	1.0–1.3
PS[5]	Polystyrene 267 kg/mol h_0=75 nm	Spherical silica D=3.7×10^{-6} m m=5.20×10^{-14} kg	500–800	0.9–2.8
PC[6]	Polycarbonate 26 kg/mol h_0=200 nm	Spherical silica D=7.6×10^{-6} m m=4.25×10^{-13} kg	504	1.7
P(VDF-TrFE)[10]	poly(vinylidene fluoride-co-trifluoroethylene) 450 kg/mol h_0=73 nm	Spherical silica D=9.2×10^{-6} m m=7.54×10^{-13} kg	900–1050	3.8
Steel[11]	304 stainless steel h_0=3×10^{-3} m	Spherical steel D=12.5×10^{-3} m m=8.4×10^{-3} kg	500–990	0.2–0.7
Aluminum[12]	h_0=1.27×10^{-3} m	Spherical steel D=6.35×10^{-3} m m=1.05×10^{-3} kg	850	0.6
Kevlar[7]	Kevlar KM2 (Polyaramid fiber woven fabric) h_0=2.0×10^{-4} m	Spherical aluminum D=5.56×10^{-3} m m=2.5×10^{-4} kg	1000–1100	0.9–1.2
Dyneema[8]	UHMWPE fiber woven fabric h_0=5.8×10^{-4} m	Cylinderical steel D=5.5×10^{-3} m m=1.0×10^{-3} kg	470–520	2.0–2.4

*Ultra-high molecular weight polyethylene

Table S2. Parameters of ballistic tests
Parameters of macroscopic and LIPIT ballistic tests with D/h_0>4 for the different materials compared in Figure 3c.

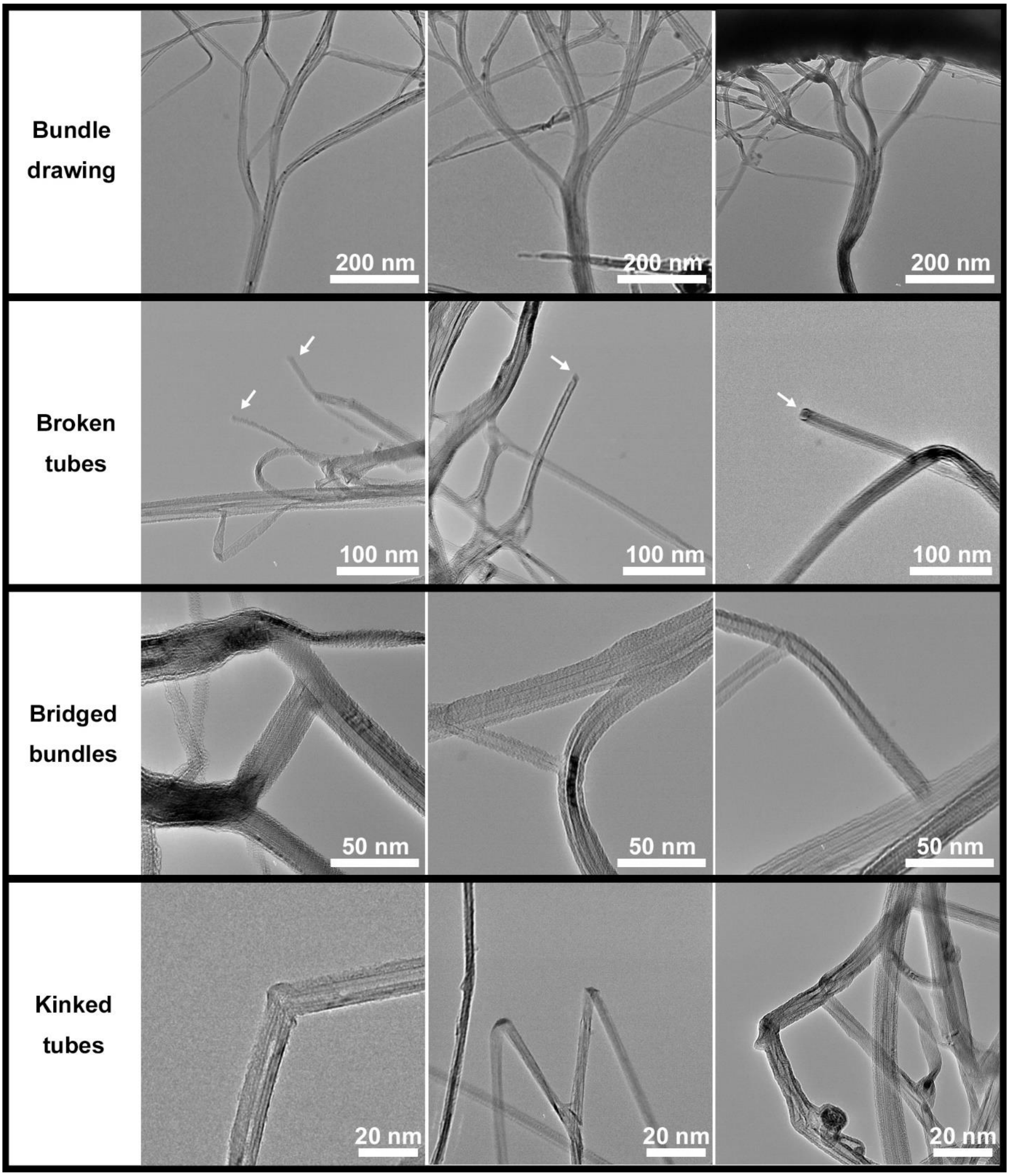

Figure S1. Tube morphologies from HRTEM of perforated regions
Top row: Local bundling/drawing of MWCNTs at the peripheral region of the impact. Second row: Fractured tubes in the perforated mat (white arrows). Third row: Covalent T junctions and π-π Y junctions of the MWCNT network. Bottom row: Localized tube kinking due to elastic snap-back after fracture.

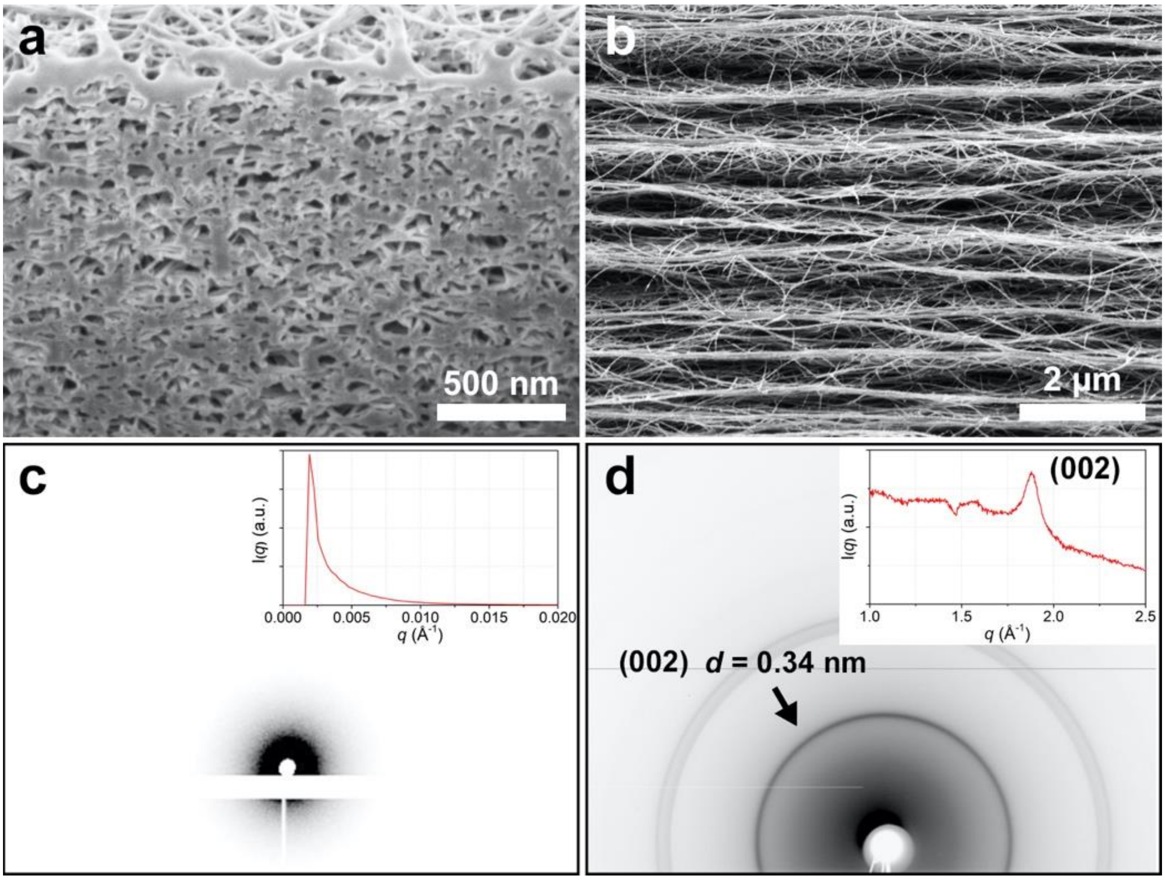

Figure S2. MWCNT mat characterization
(a) Cross sectional SEM image of the porous mat after focused ion beam milling. (b) Low magnification SEM cross sectional view of MWCNT mat prepared by cutting with a CO_2 laser. Some layers have splayed apart due to the directed air pressure. (c) 2D SAXS and (d) 2D WAXS patterns and inserted radial intensity traces indicating planar isotropic nature of tube/bundle morphology.

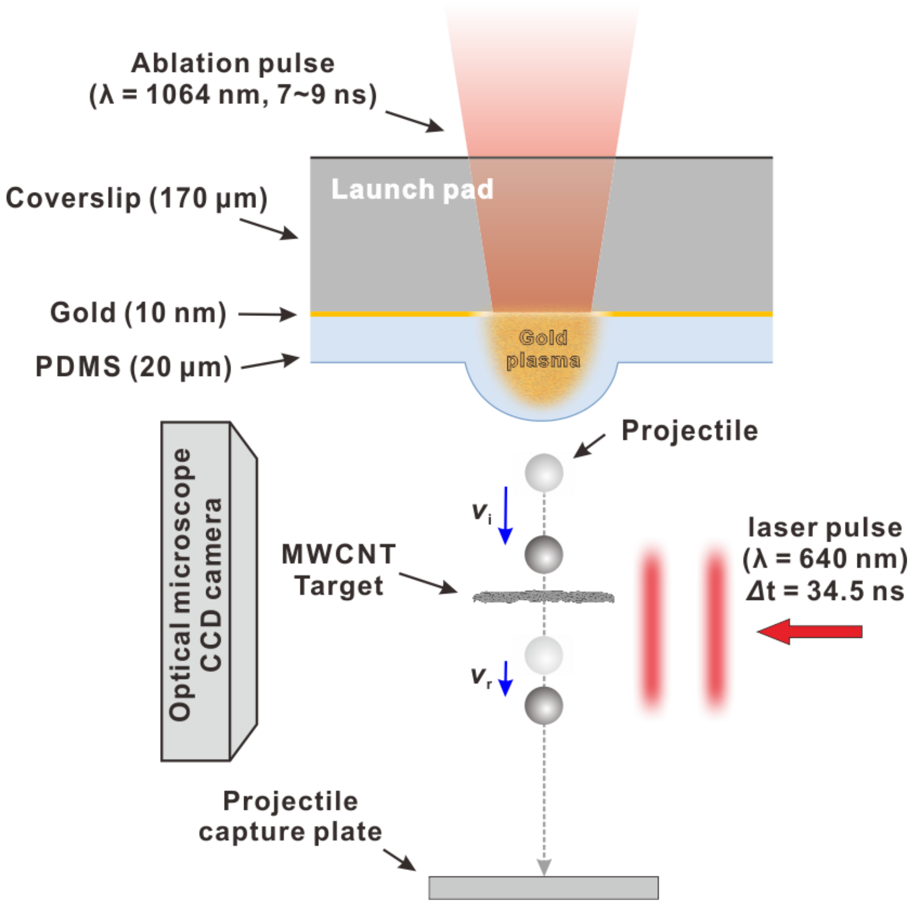

Figure S3. LIPIT apparatus schematic
A 1064 nm laser pulse is focused onto the gold film in the launch pad. The ablation of the gold creates a bubble in the PDMS film which launches the silica projectile toward the MWCNT target affixed to a TEM grid. Two 640 nm laser pulses (time interval ~34.5 ns) illuminate the moving silica micro-bullet. A CCD camera records the images to measure the in-flight projectile residual velocity. Projectiles that have perforated the target are captured on a polymer plate behind the target.

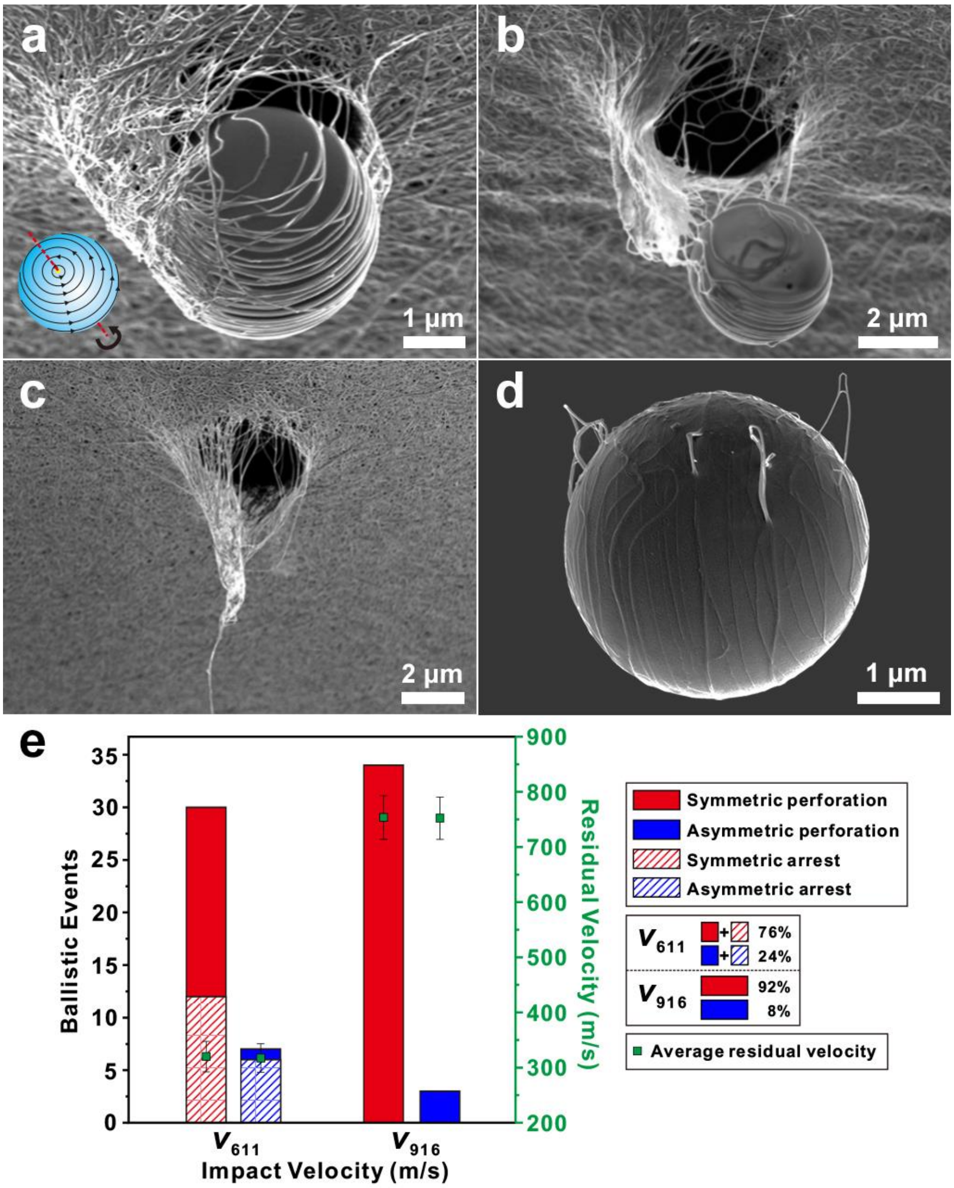

Figure S4. Impacts with projectile rotation
Morphological features associated with projectile penetration and perforation of h_{205} mats. (a) SEM images (tilted 45 degrees) of mats shot at v_{611} showing the early stages of mat deformation and creation of latitudinally aligned principal tubes. The inset schematic defines the latitudinal principal (load carrying) tubes. The distinct latitudinal wrapping by the tubes rather than the usual longitudinal orientation of the tubes on the projectile surface implies that the projectiles are undergoing rotation on passing through a mat. (b) Arrested sphere with extended fibrils. The projectile has deflected the left side of the mat forward, but at the right side, the combination of the forward and rotational motions of the sphere have extended the tubes into a parallel array with the tube directions normal to the axis of rotation (c) Perforated mat impacted at v_{611} suggesting projectile spin. (d) SEM image of captured projectile after perforation. (e) Statistical analysis of the occurrence of projectile spin during ballistic events. We estimate about 25% of the v_{611} projectiles have sufficient rotational speed to noticeably influence the deformation geometry of the mat. While the deformation texture is different, there seems to be no strong correlation with the translational KE absorbed for projectiles with significant spin.

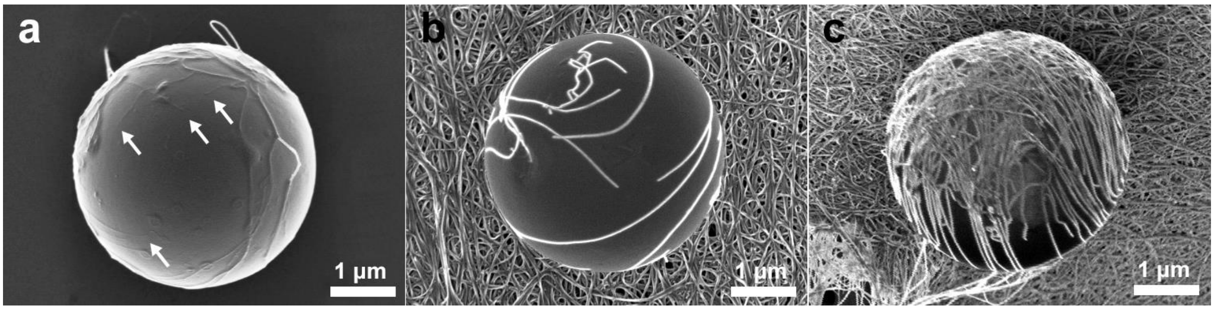

Figure S5. Tensile fractures of MWCNTs
SEM images of tubes adhering to projectiles. (a) White arrows indicate fractured tubes on a projectile on the capture plate. The gold coating to prevent charging on the polymer capture plate lowers the contrast between tubes and projectile. (b) and (c) uncoated arrested projectiles retained on the exit surface of the MWCNT mat. The nonconductive silica sphere has positive charge and appears dark compared to the bright, conductive CNTs. The high contrast shows obvious tensile fractures of principal tubes.

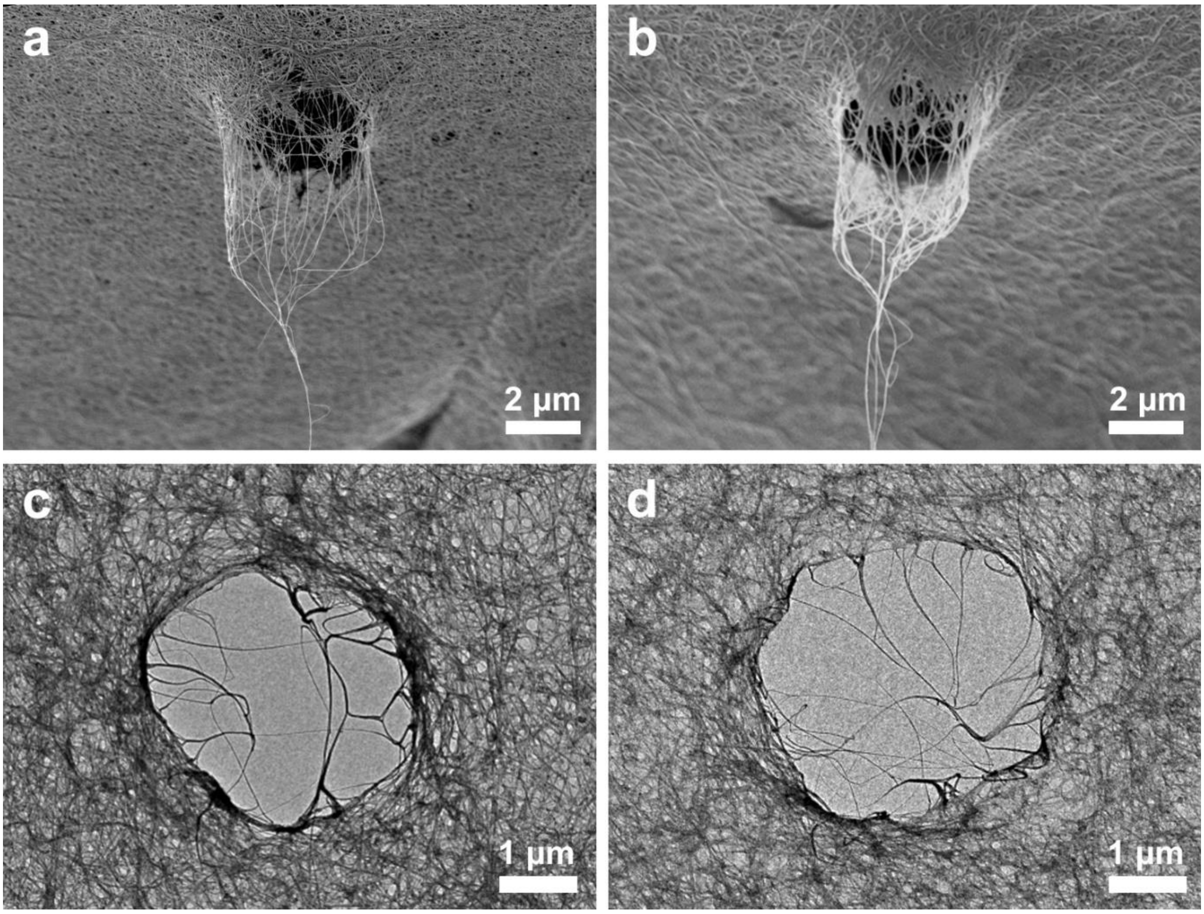

Figure S6. Axisymmetric perforation morphologies
Perforations for an incident velocity of v_{916} for a h_{205} mat. Near axisymmetric deformation of the mat from projectiles with little or no rotation. (a) and (b) 52˚ tilted view SEM images. (c) and (d) normal view BFTEM images.

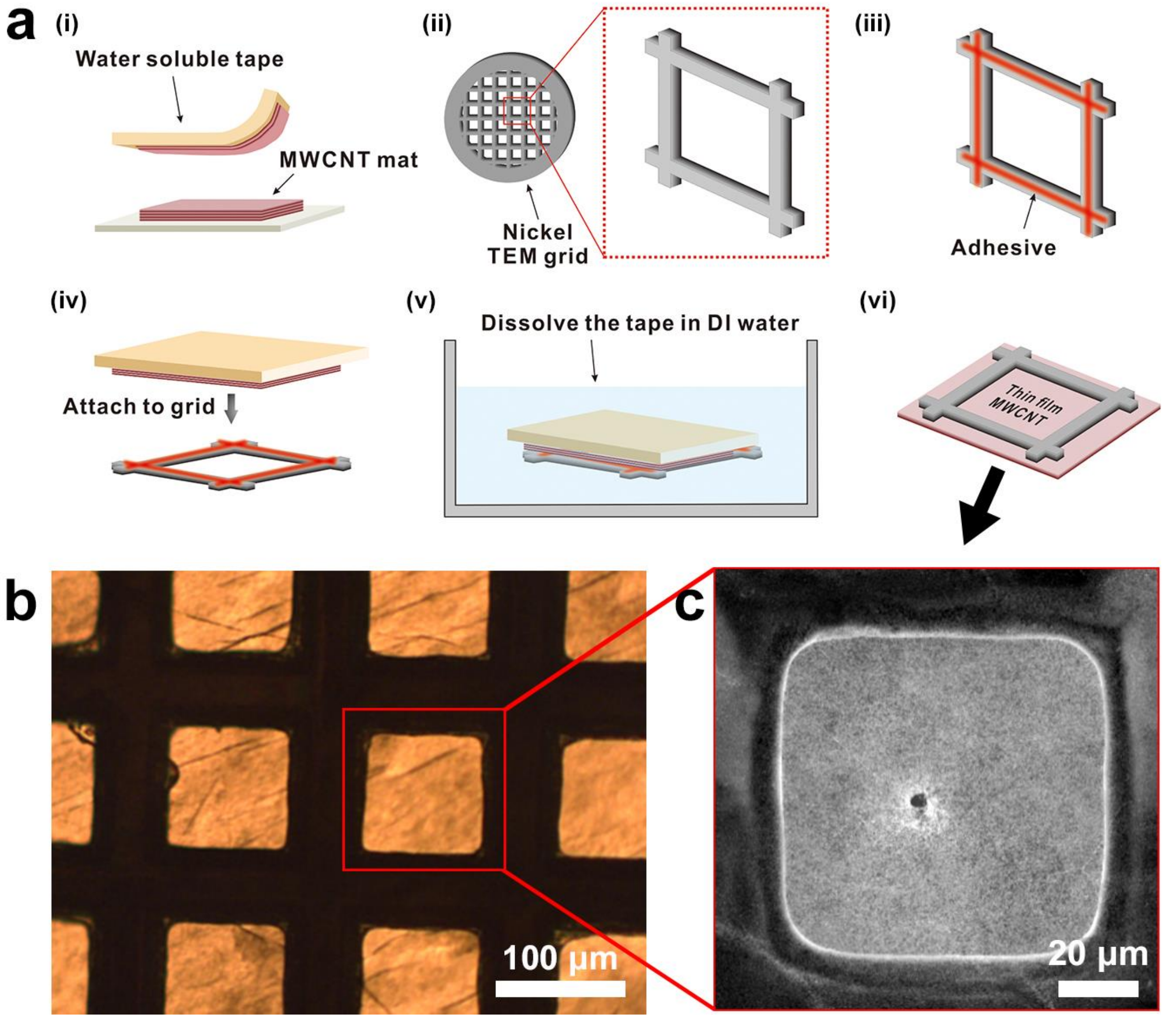

Figure S7. Sample preparation
(a) Schematic illustration of MWCNT thin film target preparation process. (b) Optical image of h_{205} mat on TEM grid. (c) Exemplary SEM image of TEM grid window covered by a mat that has been perforated by a LIPIT projectile.

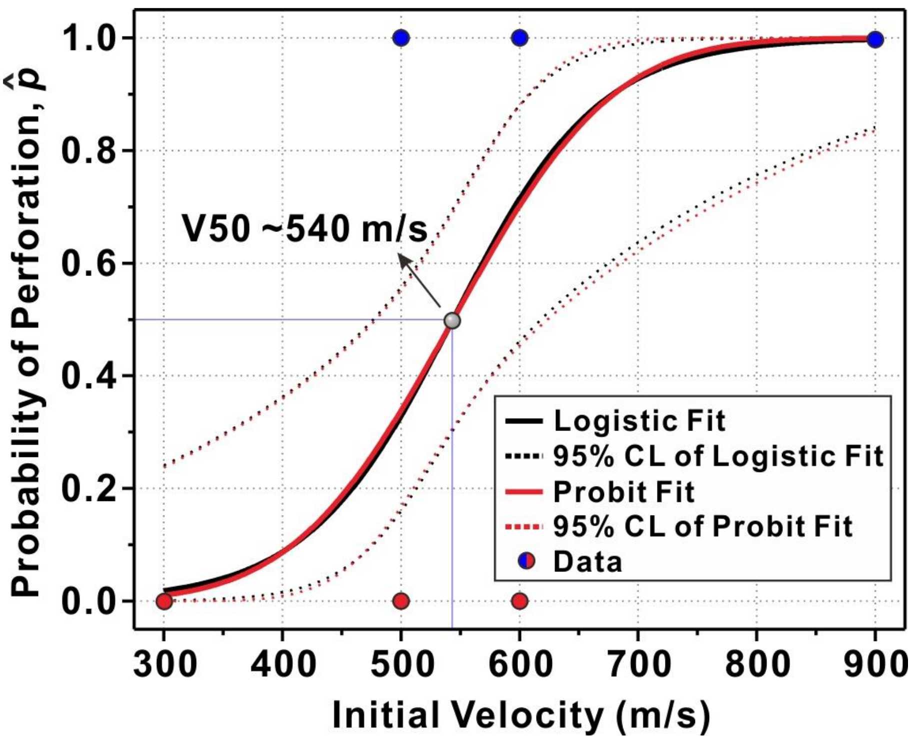

Figure S8. V50 ballistic limit
The V50 ballistic limit is an important standard for protective materials and was determined for the h_{205} mats. The probability of perforation vs. impact velocity data was fit to logistic and probit curves[13] with 95% confidence level (CL) and both fits yield a nanoscale V50 as ~540 m/s (n=51 shots).[14] The close values of the probit and logistic 95% CLs indicates that the MWCNT material displays consistent ballistic performance over many impacts.

References

[1] J.-H. Lee, P. E. Loya, J. Lou, E. L. Thomas, *Science* **2014**, *346*, 1092.

[2] K. Koziol, J. Vilatela, A. Moisala, M. Motta, P. Cunniff, M. Sennett, A. Windle, *Science* **2007**, *318*, 1892.

[3] J. J. Vilatela, J. A. Elliott, A. H. Windle, *ACS Nano* **2011**, *5*, 1921.

[4] W. Xie, R. Zhang, R. J. Headrick, L. Taylor, S. E. Kooi, M. Pasquali, S. Muftu, J.-H. Lee, *Nano Lett.* **2019**, *19*, 3519.

[5] J. Hyon, O. Lawal, O. Fried, R. Thevamaran, S. Yazdi, M. Zhou, D. Veysset, S. E. Kooi, Y. Jiao, M.-S. Hsiao, J. Streit, R. A. Vaia, E. L. Thomas, *Mater. Today* **2018**, *21*, 817.

[6] E. P. Chan, W. Xie, S. V. Orski, J.-H. Lee, C. L. Soles, *ACS Macro Lett.* **2019**, *8*, 806.

[7] Y. Park, Y. Kim, A. H. Baluch, C.-G. Kim, *International Journal of Impact Engineering* **2014**, *72*, 67.

[8] Z. Yuan, X. Chen, H. Zeng, K. Wang, J. Qiu, *Compos. Struct.* **2018**, *204*, 178.

[9] P. Morgan, *Carbon Fibers and Their Composites*, CRC press, **2005**.

[10] J. Cai, R. Thevamaran, *Nano Lett.* **2020**.

[11] J. Dean, C. Dunleavy, P. Brown, T. Clyne, *International Journal of Impact Engineering* **2009**, *36*, 1250.

[12] W. Golsdmith, S. Finnegan, *International Journal of Mechanical Sciences* **1971**, *13*, 843.

[13] J. H. Aldrich, F. D. Nelson, E. S. Adler, *Linear Probability, Logit, and Probit Models*, Sage, **1984**.

[14] R Core Team (2019). *R: A language and environment for statistical computing, R Foundation for Statistical Computing,* Vienna, Austria,URL http://www.R-project.org/.
